# Do Emotions Benefit Investment Decisions? Anticipatory Emotion and Investment Decisions in Non-professional Investors

**DOI:** 10.3389/fpsyg.2021.705476

**Published:** 2021-12-09

**Authors:** Neal S. Hinvest, Muhamed Alsharman, Margot Roell, Richard Fairchild

**Affiliations:** ^1^Department of Psychology, University of Bath, Bath, United Kingdom; ^2^School of Management, University of Bath, Bath, United Kingdom

**Keywords:** anticipatory emotion, skin conductance, skin conductance response, investment, trading, finance

## Abstract

Increasing financial trading performance is big business. A lingering question within academia and industry concerns whether emotions improve or degrade trading performance. In this study, 30 participants distributed hypothetical wealth between a share (a risk) and the bank (paying a small, sure, gain) within four trading games. Skin Conductance Response was measured while playing the games to measure anticipatory emotion, a covert emotion signal that impacts decision-making. Anticipatory emotion was significantly associated with trading performance but the direction of the correlation was dependent upon the share’s movement. Thus, anticipatory emotion is neither wholly “good” nor “bad” for trading; instead, the relationship is context-dependent. This is one of the first studies exploring the association between anticipatory emotion and trading behaviour using trading games within an experimentally rigorous environment. Our findings elucidate the relationship between anticipatory emotion and financial decision-making and have applications for improving trading performance in novice and expert traders.

## Introduction

The key to being a successful trader is a huge business. While many academics argue that emotions degrade trading performance (Gray, 1999; Lerner and Keltner, 2001; Lo et al., 2005; Lucey and Dowling, 2005; Shiv, et al., 2005; Schunk and Betsch, 2006), there are those who contest that emotions have, instead, a positive impact (Ackert et al., 2003; Ackert and Deaves, 2010).

Neoclassical economics has eschewed the investigation of emotions in favour of portraying decision-makers as “rational” and non-emotional. Newer developments in behavioural economics and emotional finance have mostly relied on a psycho-analytic approach to understand the effect of emotions on trading decisions. In their research into the dot.com bubble of the twenty-first century, Taffler and Tuckett (2005) pioneered the field of emotional finance by introducing Freud’s theory of Psychoanalysis and “phantasy” objects to investment behaviour. Taffler and Tuckett theorised that a range of unconscious emotions dictate investors’ decision-making, more than knowledge of company fundamentals or future growth potential. For example, continual growth in share price is associated with excitement and overconfidence in investors which is, in turn, associated with “herding” behaviour in groups of investors, creating market bubbles (Taffler and Tuckett, 2005; Shefrin, 2007; Taffler et al., 2017). When the bubble “bursts”, high levels of negatively valenced emotions such as regret and guilt further impact investment decisions, typically promoting risk avoidance (Taffler and Tuckett, 2005; Taffler et al., 2017). Thus, unconscious emotion significantly impacts trading behaviour.

Empirical research within psychology indicates that unconscious anticipatory emotions are critical components of a functional decision-making system (Bechara et al., 1997, 2001). Anticipatory emotions input physiological (somatic) signals of emotion into whatever decisions we are currently making, with a traditional view that they provide “gut feelings” that push us towards particular alternatives within the decision (Bechara et al., 2005). Dysfunction of brain areas involved in the formation of anticipatory emotions impacts decision-making whereby individuals may struggle to choose between seemingly simple alternatives (Damasio, 2008).

Although a critical component of decision-making, the role of anticipatory emotions remains under debate (Dunn et al., 2006). Davis et al. (2009) posit that anticipatory emotions, rather than being a rapid, coarse, signal of value or risk (the traditional, “emotions-as-input” perspective), they represent a relatively slower process (Hinson et al., 2006) interacting with cognitive processes in response to uncertainty or contextual novelty and signal a readiness to learn (the “emotions-as-output” perspective). Otto et al. (2014) support the emotions-as-output perspective, showing that anticipatory emotions interact with cognitive processes and provide reflection on choice consequences. Whatever the stance on anticipatory emotions, there is agreement that they are important signals integrated into current decision strategies. It is important to note that there appears to be a “dark side” of anticipatory emotion, where high levels of unconscious emotion can degrade choice behaviour (Shiv et al., 2005). Given the case that anticipatory emotions are not comprehensively “good” nor “bad” for investment decisions, what can we learn about the relationship between anticipatory emotion and risk-aversion/−seeking in a range of trading environments?

This study addresses this question utilising a neuroeconomic approach to measure anticipatory emotion, *via* recordings of Skin conductance response (SCR), in multiple trading games with varying share patterns. Participants with varying levels of trading experience decided how to allocate wealth between a safe, but low paying, option (the “bank”), or a potentially higher-risk, but higher-payoff, option (the share).

## Materials and Methods

### Participants

Thirty participants (18 male) were recruited with a mean age of 27.13 (*S.D.* 7.66) years. Twenty-four participants were students at the University of Bath with the remaining six participants being University employees. Preliminary analyses revealed no systematic differences between student and nonstudent responses; thus they were combined in all analyses. Eighteen participants classified themselves as Caucasian European, three as Asian, one as Afro-Caribbean and two classified their ethnicity as “other.” Eight participants reported that they had played the stock market previously. Out of these eight participants, one played daily, one did not play daily but several times per week, two played several times per month but not weekly and four played several times per year but not monthly.

Participants received £5 remuneration for their participation. To promote a motivation to perform well on the task there were also prizes of £70, £20 and £10 for the individuals who obtained the highest, second highest, and third highest overall percentage return on investment, respectively, (calculated over all games). Informed written consent from all participants was obtained. The study was approved by the Psychology Research Ethics Committee at the University of Bath.

A power analysis was performed to check the appropriateness of the sample size using the results from the multilevel analysis between anticipatory SCR and returned trial-by-trial as due to the sensitivity of the test this stage would demand the highest sample size. G^*^Power 3.1.5 (Faul et al., 2007) was used to calculate power. Based upon an R^2^ of.22 (taken from stage 1 of the analysis, see *Data Analysis* section), we computed that a sample of 30 participants yielded a power of 0.88, thus the sample size is appropriate.

### Material and Apparatus

The materials required for the experiment comprised of four stock market games (henceforth shortened to “stock games”). Physiological data were collected using a BIOPAC MP 150 system with a 500-Hz sampling rate. SCR activity was measured using a constant voltage (0.5 V) with Ag-AgCl electrodes attached to the distal phalanx of the middle and index finger of the non-dominant hand. Standardisation was achieved *via* the following steps; the SCR signal was low-pass filtered through the amplifier (1.0 Hz) and high-pass filtered (0.05 Hz) to extract the phasic SCR. A threshold of 0.02 microsiemens (μS) was used. Anticipatory SCR was extracted between the 3 s before a click to move to the next trial and 2 s after the start of the trial. SCRs are slow-wave functions and this window was used to allow capture of the peak amplitude of an anticipatory SCR that crossed the 0.02μS threshold (Dawson et al., 2011). Data were acquired in a quiet room controlled at room temperature. AcqKnowledge (version 4.3) analysis software and SPSS (v. 22) were used.

To explore the valence of emotion experienced within each game the Positive and Negative Affect Scale (PANAS) was given to all participants (Watson et al., 1988). The PANAS is a 20-item self-report questionnaire. Participants report to what level they feel 10 positive and 10 negative adjectives during the stock game that they had just experienced.

**Figure 1:**
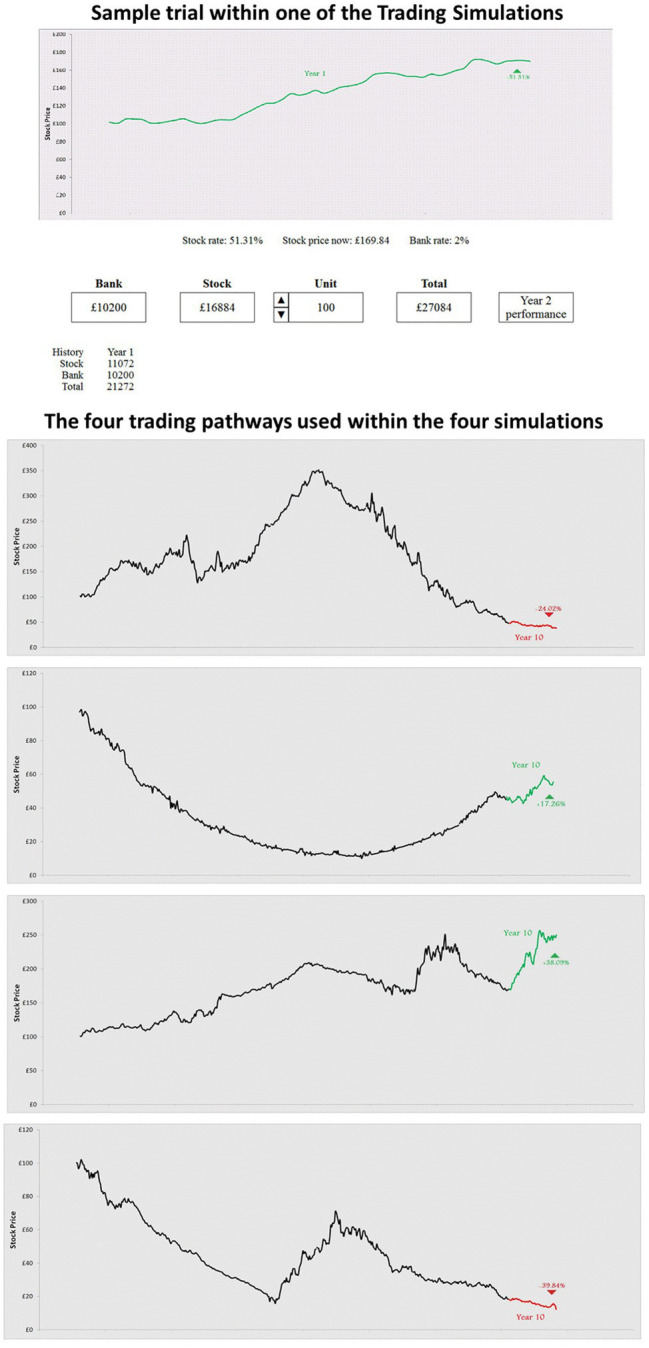
Screenshot of one trial from a stock market game and the share pathways used in the four games.

Participants were presented with four different computerised stock games. Participants were initially instructed that they had inherited £20,000, half in stocks and half in cash. Over a 10-year period (represented by 10 sequentially presented trials), they were to decide the amount they wished to invest in stock and the amount they would like to save as cash. The participants were told that their goal was to make as much money overall. In their first trial, they earned 2% interest on the cash and earned or lost money on the stocks dependent on its current price. Visual and descriptive information as to the behaviour of the stock and the amount of money they made in stocks, cash and overall was provided for each trial (see Figure 1 for an example of one trial and pathways from each game). Stock game 1 followed an “n-shaped” stock market fluctuation, stock game 2 a “u-shaped” stock market scenario, stock game 3 an “upward” fluctuation and stock game 4 a “range trading” scenario (Figure 1). Participants could respond in their own time within each trial. When the participants clicked to move onto the next trial it was immediately shown. There was a non-linear relationship between risk aversion and return on initial investment such that those who are highly risk-seeking or risk-averse will not perform as well as those at a mid-point of risk aversion (Fairchild et al., 2016).

### Procedure

Each participant first read an information sheet and gave written consent to participate. They answered a demographic questionnaire providing information pertaining to their age, sex, gender, ethnicity, educational level, degree enrolled upon (if applicable) and if they trade in stock markets and, if so, the frequency of engagement during a typical month. Participants also completed the PANAS to measure initial emotional status.

Each participant was connected to the BIOPAC to measure SCR. After fitting electrodes to their non-dominant hand, participants were instructed to keep that hand still to avoid movement artefacts within the SCR waveform. A practice stock game was presented to the participant, to assess that they had fully comprehended the task and instructions. Participants subsequently started their first stock game. The order of presentation for all four stock games was randomised between participants to prevent order effects. Participants were given the PANAS after each game and instructed to rate themselves as to their emotional experience during the stock game that they had just completed. Once the participant had finished all four stock games they were verbally debriefed.

### Data Analysis

The variables of interest related to performance were the returns (i.e. profit or loss) that each individual made and anticipatory SCR. It is pertinent to explore returns as they tell us about whether the general trend on an individual’s choice behaviour was to make a profit or loss.

For investigations into anticipatory emotion, the anticipatory SCR for each trial was associated with performance on the following trial, therefore, in each game, there were nine data points. SCR data are commonly positively skewed so a close look at the structure of the data was warranted. Any SCR values of zero (due to not reaching threshold for occurrence of a SCR) were removed from the data to avoid artificial “pushing” of the data into an extreme positive skew. Data were explored with and without outliers removed (*via* a 25/75% confidence interval threshold). There were no notable differences in skewness and kurtosis values between the two datasets so the original dataset was used in order to increase the amount of data analysed.

In order to ascertain the valence of the anticipatory emotion experienced by participants in each trend PANAS responses were coded into responses to adjectives that had a positive valence and those that had a negative valence. This gave scores on both valence for each trend. Residual PANAS scores were calculated by subtracting the value from the initial PANAS in order to control for each participant’s emotional state before playing the games. These eight scores were entered into a 2 × 4 repeated-measures ANOVA to test whether there were differences in the valence of emotion experienced within each trend and between trends.

## Results

### Relationship Between Anticipatory SCR and Returns Analysed Trial-by-Trial

Table 1 shows performance measures within each of the trends. This table shows that trends 1 and 4 were associated, on average, with losses in return, while trends 2 and 3 were associated with more overall gain on participants’ initial endowments.

**Table 1:** Performance measures for each stock game.

	Trend 1		Trend 2		Trend 3		Trend 4	
	Mean	*SD*	Mean	*SD*	Mean	*SD*	Mean	*SD*
Average return on original endowment	−5.28%	5.44%	5.83%	3.66%	5.38%	1.46%	−5.46%	6.16%
Total trading volume	£49,963.46	£31,312.61	£17,195.87	£22,496.61	£35,970.06	£23,791.26	£13,823.91	£9,423.69
Average trading volume in each period	£5,551.50	£7,849.88	£1,878.83	£6,222.53	£3,904.71	£5,488.05	£1,513.55	£2,664.54
Return of “perfect” trader on original endowment	13.96%	–	13.67%	–	13.40%	–	11.25%	–

SD stands for standard deviation. The “perfect” trader is a fictitious trader who invests all money into shares when the share price subsequently increases and invests all money into the bank when the share price subsequently decreases.

**Figure 2:**
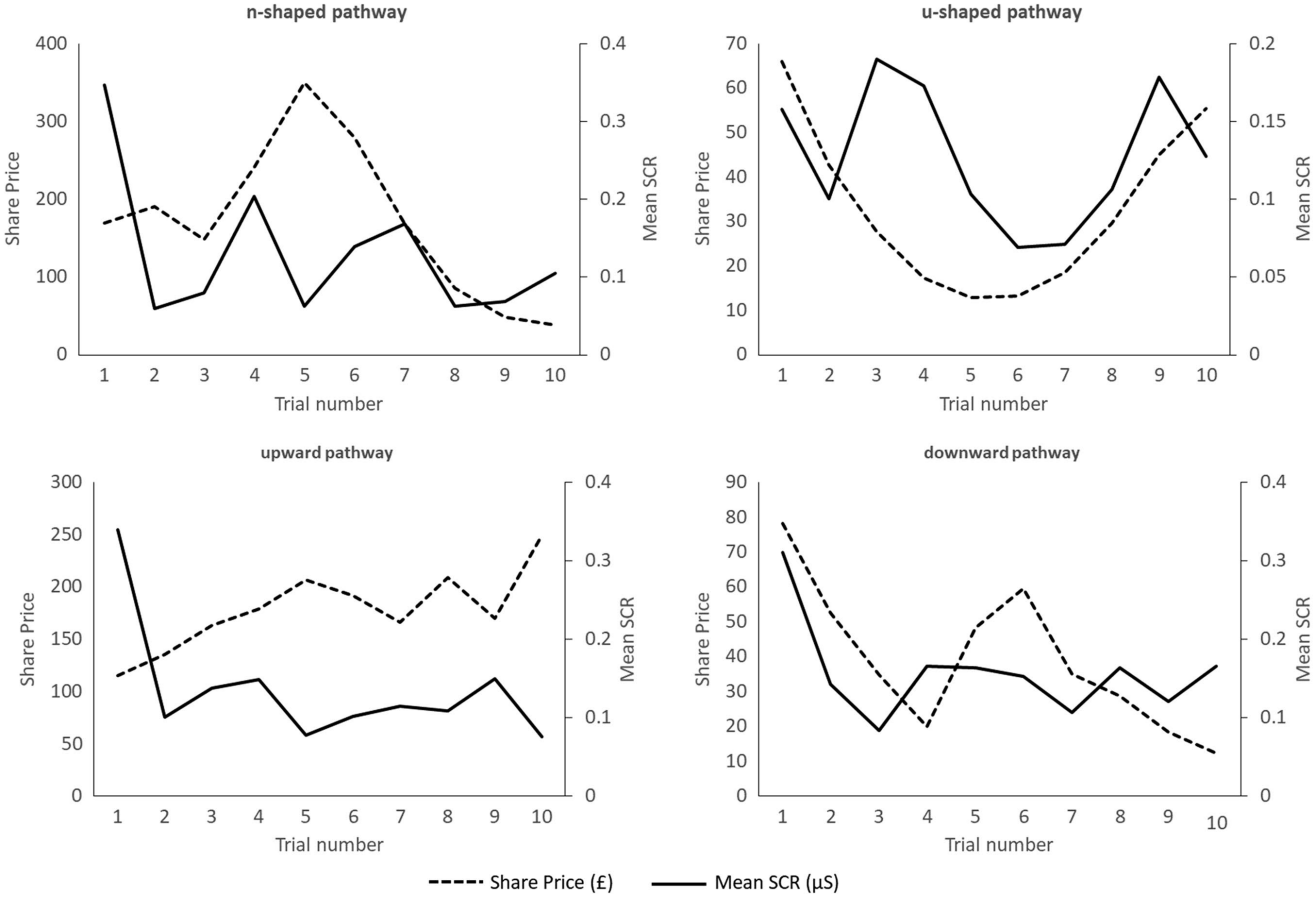
Mean trial-by-trial skin conductance response (SCR) within each game plotted alongside share price.

Multilevel modelling (panel data) was used to correlate anticipatory emotion with return at each time point in each game. Return in this analysis was calculated as the percentage gain or loss in one trial *vs.* the previous. Multilevel modelling permitted the exploration of how anticipatory emotion was associated with decision-making within each time point compared to aggregating the data, permitting a fine-grained analysis of investor behaviour. Figure 2 shows the mean SCR and percentage return per trial in all four games.

For game 1 (n-shaped), greater anticipatory SCR was associated (an almost significant correlation) with improved performance, OR (odds ratio) = 0.03, *p* = 0.06, R^2^ = 0.001. This means that when experiencing n-shaped trends greater levels of anticipatory SCR is associated with small, but potentially meaningful, improvements in investment performance. In game 2, there was a significant inverse correlation between anticipatory SCR and returns per trial, OR = −0.18, *p* = 0.039, R^2^ = 0.22. There was no significant correlation between returns and SCR in game 3, OR = 0.09, *p* = 0.43, R^2^ = 0.003, and game 4, OR = 0.03, *p* = 0.77, R^2^ = 0.0004.

### Exploration of Anticipatory Emotion Within Upward or Downward Share Sub-trends Within Games

Upward and downward trends in games 1 and 2 were extracted, and multilevel analysis of the data was performed as in the above section. This was conducted in order to explore whether performance improvements/degradations could be associated with simple linear responses to upward or downward trends or whether it was a response to the amalgamation of upward and downward trends in each game. There were no significant correlations between anticipatory SCR and return in any of the sub-trends, suggesting that performance improvements/degradations seen in the n-shaped and u-shaped trends were not down to a simple response to upward/downward trends but a response to the trend as a whole.

### The Effect of a Previous Outcome for an Individual on Anticipatory Emotion for a Subsequent Choice

To investigate how the outcome from a previous investment choice is associated with anticipatory emotion for a subsequent investment choice we used multi-level modelling to correlate the amount of return following a choice (which would be a value of a gain or loss) shown at the start of a trial with the SCR within the anticipatory window at the end of the same trial (i.e. anticipatory emotion associated with choice after feedback has been processed). For each game, we assessed those outcomes that ended in gain and, separately, those that ended in loss. Anticipatory emotion was not significantly correlated with preceding gain or loss amount, thus anticipatory emotion appears yoked to the current decision event and not previously experienced gains or losses.

### Overall, Consciously Reported, Emotional Reaction to Each Simulation

**Figure 3:**
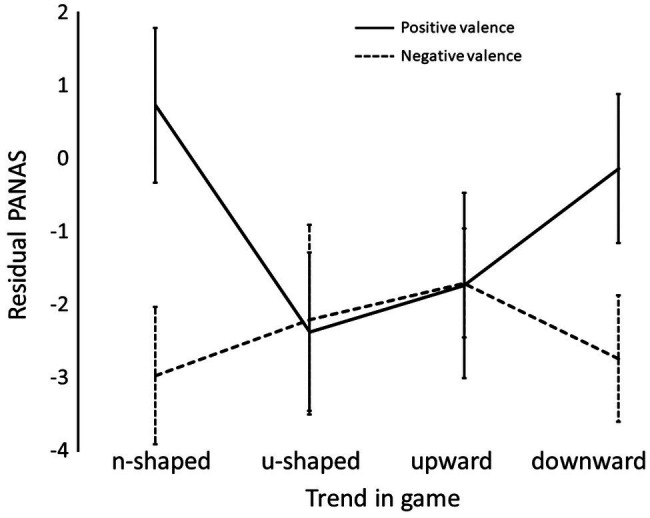
Mean positive and negatively valence residual positive and negative affect scale (PANAS) scores within each game. Error bars show 95% confidence intervals.

Mean residual PANAS scores separated by game and emotional valence are shown in Table 2. There was no significant difference in the level of positive, compared to negative, emotion reported after the games, *F*(1,29) = 1.67, *p* = 0.21, η^2^ = 05. The magnitude of emotion reported after each game was also, overall, not significantly different, *F*(3,87) = 1.53, *p* = 0.21, η^2^ = 05. There was a significant interaction, *F*(3,87) = 3.66, *p* = 0.016, η^2^ = 0.11. The interaction arose from equal levels of reported emotion in each valence in games 2 and 3 but much higher levels of negative compared to positive consciously reported emotion in games 1 and 4 (Figure 3). N.B. Significant results from simple effects are shown (^*^ = *p* < 0.05; ^**^ = *p* < 0.001). Effects shown at the top of the figure relate to positive valence and that on the bottom refers to negative valence.

**Table 2:** Average residual PANAS scores for each game.

		Mean	95% Confidence interval
Positive valence	Trend 1	0.73	2.11
	Trend 2	−2.37	2.17
	Trend 3	−1.73	2.54
	Trend 4	−0.13	2.04
Negative valence	Trend 1	−2.97	1.89
	Trend 2	−2.20	2.60
	Trend 3	−1.70	1.50
	Trend 4	−2.73	1.73

Residual scores were calculated by subtracting the value of reported positive/negative valence emotion after each game and subtracting it from the baseline positive/negative PANAS score, respectively, reported before the participant played the games.

## Discussion

The key finding within this study is that the relationship between anticipatory emotion and choice behaviour is dependent on context, namely the share movement. In our games, trading gains acquired within an n-shaped share trend were associated with higher levels of anticipatory emotion, but in a u-shaped trend, gains were associated with lower levels of anticipatory emotion.

Our findings that the association between anticipatory emotion and trading performance in context-dependent is supported by Shiv et al. (2005). In this study, patients with damage to the ventromedial frontal cortex, who exhibit blunted anticipatory SCRs, and healthy participants were gifted $20 and given 20 opportunities to invest subsequent $1 portions of that money into a 50/50 gamble between losing $1 or winning $2.50. Expected utility demands that the best option is to gamble with all $1 portions. However, compared to 79% of patients who gambled, only 58% of healthy participants gambled. For healthy decision-makers, an injection of emotion into the decision as to whether to gamble led to a heightened level of risk aversion. This further supports the conclusion that anticipatory emotions will not lead to broad improvements in performance. Our study is novel in that it extends this finding to richer games of trading behaviour.

**Figure 4:**
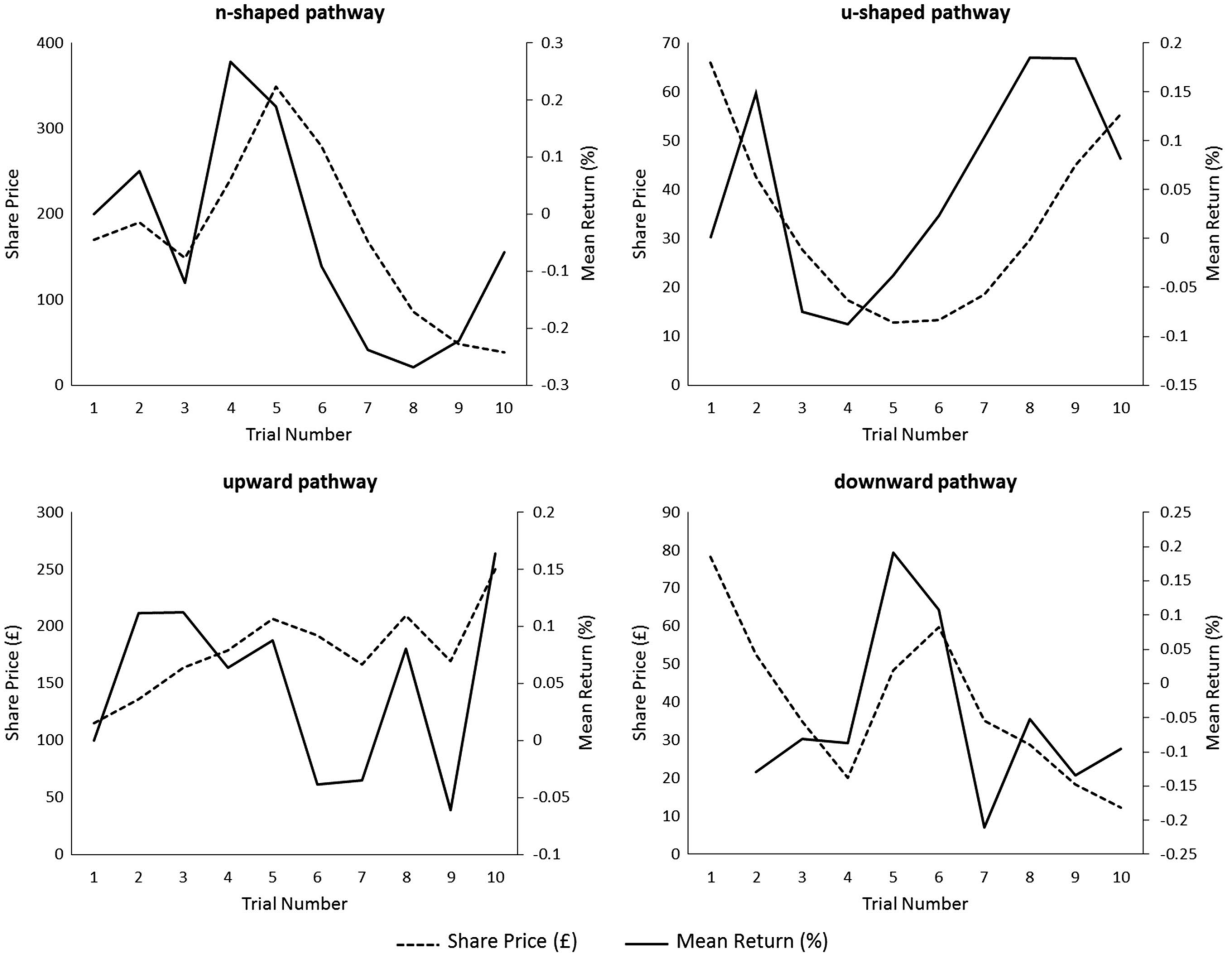
Comparison of mean return on investment to the share pathway on the four stock market games.

We are significantly more likely to be risk-averse when outcomes are framed in terms of what we could gain compared to what could be lost (Kahneman and Tversky, 1979; Tversky and Kahneman, 1981). In the n-shaped frame, participants experience an upward (gain) trend followed by a downward (loss) trend. In the u-shaped trend, participants experience a downward (loss) trend followed by an upward (gain) trend. It is pertinent to note that returns commonly followed the share pattern in all games, so participants typically experienced gain or loss aligned with an increase or decrease in share price, respectively (Figure 4). The evidence for framing effects can be noted from the analysis of the PANAS whereby the patterns associated with increased losses, games 1 and 4, were associated with higher levels of negative compared to positive reported emotion compared to patterns associated with gains, games 2 and 3, where the magnitude of reported negative and positive emotions was approximately equal. Further evidence for framing effects can be taken from part 4 of the analysis where upward and downward sub-trends in the n- and u-shaped games were extracted. There was no simple linear effect between SCR and the sub-trends, and therefore, significant results are a product of the entire share pattern. This may explain the non-significant results for game 3 (upward share pattern) and game 4, (downward share pattern) in which, although framing effects may occur, they may not be as salient as in games 1 and 2. In games 1 and 2, the participant was faced with a situation where participants tended to have a “winning streak” followed by greater losses or by turning around a “losing streak” into a positive return. In games 3 and 4, the share pattern was either upward or downward in nearly all trials. Agency, or responsibility for outcomes based on a person’s choices, may be higher in games 1 and 2 where a win changed to a loss, or *vice versa*, compared to games 3 and 4 where the decision-maker could predict with greater accuracy the share’s pattern. This is potentially related to Zeelenberg et al. (1998) where participants experienced different emotions when they experienced greater agency in instances where their own decisions ended in loss (leading to a more visceral feeling of regret) *vs.* instances where there was no agency (leading to a less visceral feeling of disappointment). Findings may also be related to Duclos (2015) whereby the shape of a graphical trend of a stock price at the end of trading (upwards or downwards) would affect risk behaviour within subsequent investing decisions. Taken together, the shaping of the graph creates particular frames to which the investor differs to in response. An interesting next step may be to see whether the same results are found with different visual interfaces of the trading data. Previous research suggests that presentation of the same information in different visual formats, such as a graph or table, leads to differing levels of attention and processing of the financial information contained within (Ceravolo et al., 2019).

Emotions affect susceptibility to framing effects at a conscious (Covey, 2014; Lecheler et al., 2015) and unconscious (Ring, 2015) level. Furthermore, different frames engage different decision inputs within the brain. Hinvest et al. (2014) found that although a unitary brain system was involved in risky decisions regardless of framing, the frame itself elicited varying levels of activity in different neural regions within that system. Specifically, cognitive and emotional mechanisms have different levels of input into decision-making across different frames. Thus, in the current study, we feasibly conclude that the different share patterns (frames) receive different levels of input from cognitive and emotional systems leading to different patterns of emotional arousal and decision-making performance. In a potential future study, the feedback-related negativity (FRN) could be measured after each choice in the gain and loss portions of each trend to elucidate how emotion affects integration of feedback into future decision strategies as the strength of the FRN is impacted by current emotional state (Zhao et al., 2016; Gu et al., 2017).

Our results support the “emotions-as-output” hypothesis regarding the function of anticipatory emotion signals, albeit tentatively. The “emotions-as-input” hypothesis posits anticipatory SCR to be a signal of value that is based upon previous experience (Davis et al., 2009). The emotions-as-output hypothesis postulates that anticipatory SCR is a response to uncertainty and signals a need to learn. Our results indicate that anticipatory SCR is not predicted by the magnitude of gain or loss on a previous trial thus providing no evidence of a link between anticipatory SCR and previous outcomes refuting the assumptions of the emotions-as-input hypothesis.

Increasing the effectiveness of trading behaviour is big business, with a vast host of companies and websites aiming to offer support in developing an individual to make more money trading. The effect of emotions on trading performance is a common theme within this training. Many of these approaches are only loosely based on valid empirical research. Thus, research into how emotion effects trading performance is highly lucrative and essential to inform effective training. The current study supports and extends previous empirical work in this area. Lo and Repin (2002); Lo et al. (2005) and Fenton-O’Creevy et al. (2012) measured a range of psychophysiological signals, including SCR and heart rate variability, in professional traders in live trading environments and found that characteristics of the trading environment such as making positive returns and market volatility were associated with significant changes to arousal state. Interestingly, the arousal was positively associated with amount of trading experience (Lo and Repin, 2002). Experienced traders, it seems, do not “switch off” emotion but are more able to regulate their emotions and turn felt emotions into positive strategies (Fenton-O’Creevy et al., 2011, 2012). Our results extend the above findings though several means. Firstly, the current experiment explores the relationship between emotion and trading in a controlled, empirical, manner; a need highlighted by Lo and Repin (2002). Secondly, our study explores anticipatory emotions rather than those broadly felt alongside market events. This approach permits us to make inferences about how anticipatory emotion integrated into current decision strategies affect returns, a critical consideration if we wish to make inferences about how emotions are associated with actual trading decision performance. With the rise of online trading platforms (e.g. MetaTrader), a logical next step is to measure psychophysical and behavioural factors while investors engage with these platforms, potentially, with their own funds to further increase ecological validity. However, there are methodological hurdles to overcome in this approach, not least timing synchronisation between events shown *via* the online platform and psychophysical recording software and hardware.

Emotion regulation strategies designed to minimise variability in emotion have been found to increase the optimality of trading decisions (Fenton-O’Creevy et al., 2011; Hariharan et al., 2015). Our study adds novel ground to this research by suggesting that emotion regulation strategies should be yoked to the current share trend, e.g. an emotion regulation strategy when experiencing a downward trend may need to focus on maintaining high levels of arousal whereas maintaining a controlled low level of arousal will be important in an upward trend. The literature indicates that such rapid self-regulation is possible and effective in changing behaviour through simple cues, e.g. a simple command to “increase” “decrease” or “not regulate” emotions every 6.5 s with 2.5 s to “relax” between events, with participants choosing their own methods for doing so (Baur et al., 2015; Koch et al., 2018). Our study is a first step to understanding how emotion regulation strategies could be designed to be more effective. Our games are more controlled and shorter in duration to the typical real-world trading environment and further studies should present more trends and extend these games into providing longer test periods and testing of rapid self-regulation strategies.

Systems that measure SCR in traders and interrupt them when their level of arousal increases beyond a pre-determined threshold that signal high stress have been introduced as possible means of increasing trading performance (Dang et al., 2011). Our findings add to the literature to suggest that systems such as these could be extended to monitor the current share trend in addition to the individual’s unconscious emotional status and align the two in such a way that performance is maximised using the enhanced emotion regulation strategies put forward in the previous paragraph. This would necessitate development of psycho/neuro-physical methods of measurement that can identify the valence and magnitude of emotion. There is emerging work that EEG could be used to identify whether an individual is in a positive or negative emotional state which, alongside SCR, would provide measurements of both an individual’s emotional valence and level of arousal (Petrantonakis and Hadjileontiadis, 2010; Kim et al., 2013). Some of the Authors are already working in this area.

The current study has found that unconscious anticipatory emotion is associated with trading performance. Critically, the relationship between anticipatory emotion and performance is context-dependent, with greater anticipatory emotion associated with improved returns in some share patterns but negatively impact in other patterns due to a discovered link between anticipatory emotion and risk aversion. This work has implications for understanding the effect of emotions on trading performance and the design of emotional training regimes designed to improve financial returns from trading.

## Data Availability Statement

The raw data supporting the conclusions of this article will be made available by the authors, without undue reservation, upon request to the corresponding author.

## Ethics Statement

The studies involving human participants were reviewed and approved by Psychology Research Ethics Committee, University of Bath. The patients/participants provided their written informed consent to participate in this study.

## Author Contributions

NH led the development of the paper and co-supervised the research. RF co-supervised the research and contributed to drafts. MA led the analysis of data and contributed to drafts. MR collected data and contributed to analysis. All authors contributed to the article and approved the submitted version.

## Conflict of Interest

The authors declare that the research was conducted in the absence of any commercial or financial relationships that could be construed as a potential conflict of interest.

## Publisher’s Note

All claims expressed in this article are solely those of the authors and do not necessarily represent those of their affiliated organizations, or those of the publisher, the editors and the reviewers. Any product that may be evaluated in this article, or claim that may be made by its manufacturer, is not guaranteed or endorsed by the publisher.

## References

[ref1] Ackert L. K. Church B. Deaves R. (2003). Emotion and financial markets. Fed. Reserve Bank. Atlanta Econ. Rev. 88, 33–41.

[ref2] Ackert L. Deaves R. (2010). Psychology, Decision-Making and Markets. Ohio: Cengage Publishers.

[ref3] Baur R. Conzelmann A. Wieser M. J. Pauli P. (2015). Spontaneous emotion regulation: differential effects on evoked brain potentials and facial muscle activity. Int. J. Psychophysiol. 96, 38–48. doi: 10.1016/j.ijpsycho.2015.02.022, PMID: 25715271

[ref4] Bechara A. Damasio H. Tranel D. Damasio A. (1997). Deciding advantageously before knowing the advantageous strategy. Science 275, 1293–1295. doi: 10.1126/science.275.5304.1293, PMID: 9036851

[ref5] Bechara A. Damasio H. Tranel D. Damasio A. (2005). The Iowa gambling task and the somatic marker hypothesis: some questions and answers. Trends Cogn. Sci. 9, 159–162. doi: 10.1016/j.tics.2005.02.002, PMID: 15808493

[ref6] Bechara A. Dolan S. Denburg N. Hindes A. Anderson S. Nathan P. (2001). Decision-malting deficits, linked to a dysfunctional ventromedial prefrontal cortex, revealed in alcohol and stimulant abusers. Neuropsychologia 39, 376–389. doi: 10.1016/S0028-3932(00)00136-6, PMID: 11164876

[ref7] Ceravolo M. G. Farina V. Fattobene L. Leonelli L. (2019). Presentational format and financial consumers’ behaviour: an eye-tracking study. Int. J. Bank Mark. 37, 821–837. doi: 10.1108/IJBM-02-2018-0041

[ref8] Covey J. (2014). The role of dispositional factors in moderating message framing effects. Health Psychol. 33, 52–65. doi: 10.1037/a0029305, PMID: 22924446

[ref10] Damasio A. (2008). Descartes’ Error: Emotion, Reason and the Human Brain. BMJ 310:1213. doi: 10.1136/bmj.310.6988.1213

[ref11] Dang T. L. Liu S. K. Fels S. (2011). “ZenTrader, an emotion-reactive Interface.” in Proceedings of Entertainment Computing – ICEC 2011: 10th International Conference, ICEC 2011, Vancouver, Canada, October 5–8, 2011. eds. Anacleto J. C. Graham S. F. N. Kapralos B. El-Nasr M. S. Stanley K. (Berlin, Heidelberg: Springer Berlin Heidelberg), 294–299.

[ref12] Davis T. Love B. Maddox W. (2009). Anticipatory emotions in decision tasks: covert markers of value or attentional processes? Cognition 112, 195–200. doi: 10.1016/j.cognition.2009.04.002, PMID: 19428002 PMC2735832

[ref13] Dawson M. Schell A. Courtney C. (2011). The skin conductance response, anticipation, and decision-making. J. Neurosci. Psychol. Econ. 4:111. doi: 10.1037/a0022619

[ref14] Duclos R. (2015). The psychology of investor behaviour: (De)biasing financial decision-making one graph at a time. J. Consum. Psychol. 25, 317–325. doi: 10.1016/j.jcps.2014.11.005

[ref15] Dunn B. D. Dalgleish T. Lawrence A. D. (2006). The somatic marker hypothesis: a critical evaluation. Neurosci. Biobehav. Rev. 30, 239–271. doi: 10.1016/j.neubiorev.2005.07.001, PMID: 16197997

[ref16] Fairchild R. J. Hinvest N. S. Alsharman M. (2016).Warning: Trading Can Be Hazardous to Your Wealth! (Just Watch Out for Bears!) *SSRN*. Available at: https://ssrn.com/abstract=2825902 (Accessed June 01, 2020).

[ref17] Faul F. Erdfelder E. Lang A. Buchner A. (2007). G^*^power 3: A flexible statistical power analysis program for the social, behavioral, and biomedical sciences. Behav. Res. Methods 39, 175–191. doi: 10.3758/BF03193146, PMID: 17695343

[ref18] Fenton-O’Creevy M. Lins J. Vohra S. Richards D. Davies G. Schaaff K. (2012). Emotion regulation and trader expertise: heart rate variability on the trading floor. J. Neurosci. Psychol. Eco. 5, 227–237. doi: 10.1037/a0030364

[ref19] Fenton-O’Creevy M. Soane E. Nicholson N. Willman P. (2011). Thinking, feeling and deciding: the influence of emotions on the decision making and performance of traders. J. Organ. Behav. 32, 1044–1061. doi: 10.1002/job.720

[ref20] Gray J. (1999). A bias toward short-term thinking in threat-related negative emotional states. Personal. Soc. Psychol. Bull. 25, 65–75. doi: 10.1177/0146167299025001006

[ref21] Gu R. Feng X. Broster L. Yuan L. Xu P. Luo Y. (2017). Valence and magnitude ambiguity in feedback processing. Brain. Behav. 7:e00672. doi: 10.1002/brb3.672, PMID: 28523218 PMC5434181

[ref22] Hariharan A. Adam M. Astor P. Weinhardt C. (2015). Emotion regulation and behavior in an individual decision trading experiment: insights from psychophysiology. J. Neurosci. Psychol. Eco. 8, 186–202. doi: 10.1037/npe0000040

[ref23] Hinson J. Whitney P. Holben H. Wirick A. (2006). Affective biasing of choices in gambling task decision making. Cognit. Affective. Behav. Neurosci. 6, 190–200. doi: 10.3758/CABN.6.3.190 17243355

[ref24] Hinvest N. Brosnan M. Rogers R. Hodgson T. (2014). fMRI evidence for procedural invariance underlying gambling preference reversals. J. Neurosci. Psychol. Eco. 7, 48–63. doi: 10.1037/npe0000007

[ref25] Kahneman D. Tversky A. (1979). Prospect theory: an analysis of decision under risk. Econometrica 47, 263–291. doi: 10.2307/1914185

[ref26] Kim M. K. Kim M. Oh E. Kim S. P. (2013). A review on the computational methods for emotional state estimation from the human EEG. Comput. Math. Methods Med. 2013:573734. doi: 10.1155/2013/573734, PMID: 23634176 PMC3619694

[ref29] Koch S. B. J. Mars R. B. Toni I. Roelofs K. (2018). Emotional control, reappraised. Neurosci. Biobehav. Rev. 95, 528–534. doi: 10.1016/j.neubiorev.2018.11.003, PMID: 30412701

[ref31] Lecheler S. Bos L. Vliegenthart R. (2015). The mediating role of emotions: news framing effects on opinions about immigration. J. Mass Commun. Q. 92, 812–838. doi: 10.1177/1077699015596338

[ref32] Lerner J. Keltner D. (2001). Fear, anger, and risk. J. Pers. Soc. Psychol. 81, 146–159. doi: 10.1037/0022-3514.81.1.146, PMID: 11474720

[ref33] Lo A. Repin D. (2002). The psychophysiology of real-time financial risk processing. J. Cogn. Neurosci. 14, 323–339. doi: 10.1162/089892902317361877, PMID: 11970795

[ref34] Lo A. Repin D. Steenbarger B. (2005). Fear and greed in financial markets: a clinical study of day-traders. Am. Econ. Rev. 95, 352–359. doi: 10.1257/000282805774670095

[ref35] Lucey B. Dowling M. (2005). The role of feelings in investor decision-making. J. Econ. Surv. 19, 211–237. doi: 10.1111/j.0950-0804.2005.00245.x

[ref36] Otto A. Knox W. Markman A. Love B. (2014). Physiological and behavioral signatures of reflective exploratory choice. Cogn. Affective. Behav. Neurosci 14, 1167–1183. doi: 10.3758/s13415-014-0260-4 24664860

[ref37] Petrantonakis P. C. Hadjileontiadis L. J. (2010). Emotion recognition from EEG using higher order crossings. IEEE Trans. Inf. Technol. Biomed. 14, 186–197. doi: 10.1109/TITB.2009.2034649, PMID: 19858033

[ref38] Ring P. (2015). The framing effect and skin conductance responses. Front. Behav. Neurosci. 9:199. doi: 10.3389/fnbeh.2015.00188, PMID: 26300747 PMC4525055

[ref39] Schunk D. Betsch C. (2006). Explaining heterogeneity in utility functions by individual differences in decision modes. J. Econ. Psychol. 27, 386–401. doi: 10.1016/j.joep.2005.08.003

[ref40] Shefrin H. (2007). Behavioural Corporate Finance: Decisions that Create Value. Boston: McGraw-Hill International Edition.

[ref41] Shiv B. Loewenstein G. Bechara A. Damasio H. Damasio A. R. (2005). Investment behavior and the negative side of emotion. Psychol. Sci. 16, 435–439. doi: 10.1111/j.0956-7976.2005.01553.x, PMID: 15943668

[ref42] Taffler R. Spence C. Eshraghi A. (2017). Emotional economic man: calculation and anxiety in fund management. Accounting. Organizations. Soc 61, 53–67. doi: 10.1016/j.aos.2017.07.003

[ref43] Taffler R. J. Tuckett D. (2005). *A Psychoanalytical Interpretation of* *dot.com* *Stock Valuations*. SSRN. Available at: https://ssrn.com/abstract=676635 (Accessed June 01, 2020).

[ref44] Tversky A. Kahneman D. (1981). The framing of decisions and the psychology of choice. Science 211, 453–458. doi: 10.1126/science.7455683, PMID: 7455683

[ref45] Watson D. Clark L. A. Tellegen A. (1988). Development and validation of brief measures of positive and negative affect: the PANAS scales. J. Pers. Soc. Psychol. 54, 1063–1070. doi: 10.1037/0022-3514.54.6.1063, PMID: 3397865

[ref48] Zeelenberg M. van Dijk W. W. Manstead A. S. R. (1998). Reconsidering the relation between regret and responsibility. Organ. Behav. Hum. Decis. Process. 74, 254–272. doi: 10.1006/obhd.1998.2780 9719654

[ref47] Zhao D. Gu R. Tang P. Yang Q. Luo Y. J. (2016). Incidental emotions influence risk preference and outcome evaluation. Psychophysiology 53, 1542–1551. doi: 10.1111/psyp.12694, PMID: 27354122

